# 3D Printed Drug Delivery Systems Based on Natural Products

**DOI:** 10.3390/pharmaceutics12070620

**Published:** 2020-07-03

**Authors:** Ángela Aguilar-de-Leyva, Vicente Linares, Marta Casas, Isidoro Caraballo

**Affiliations:** Department of Pharmacy and Pharmaceutical Technology, University of Seville, 41012 Seville, Spain; aguilardeleyva@us.es (Á.A.-d.-L.); vlinares@us.es (V.L.); caraballo@us.es (I.C.)

**Keywords:** 3D printing, natural products, bioinks, printability, drug delivery

## Abstract

In the last few years, the employment of 3D printing technologies in the manufacture of drug delivery systems has increased, due to the advantages that they offer for personalized medicine. Thus, the possibility of producing sophisticated and tailor-made structures loaded with drugs intended for tissue engineering and optimizing the drug dose is particularly interesting in the case of pediatric and geriatric population. Natural products provide a wide range of advantages for their application as pharmaceutical excipients, as well as in scaffolds purposed for tissue engineering prepared by 3D printing technologies. The ability of biopolymers to form hydrogels is exploited in pressure assisted microsyringe and inkjet techniques, resulting in suitable porous matrices for the printing of living cells, as well as thermolabile drugs. In this review, we analyze the 3D printing technologies employed for the preparation of drug delivery systems based on natural products. Moreover, the 3D printed drug delivery systems containing natural products are described, highlighting the advantages offered by these types of excipients.

## 1. Introduction

Three-dimensional (3D) printing, also known as additive manufacturing, is gaining interest, due to its versatility, ease of use and its huge variety of applications among different fields [1,2,3,4]. Originally, it was thought as a technique to create new prototypes from new designed products (rapid prototyping) [5]. It has been applied in different industries such as ceramic, wood, plastic and metal [6,7,8]. Tissue engineering and regenerative medicine has also been interested on 3D printing development, because of their advantages for personalized medicine: sophisticated and tailor-made structures with complex designs and drug dose optimization, considering the age, gender, weight, state of the disease and genetic profile. Drug dose optimization is interesting, especially for pediatric and geriatric patients whose physiological requirements are even more specific [9,10,11]. Currently, splitting tablets by hand, or using tablets splitters is a common practice in order to obtain the appropriate drug dose content. However, these procedures are inaccurate, becoming especially dangerous for drugs with narrow therapeutic windows [12,13].

Three-dimensional printing is a process for making a physical object from a digital design by laying down successive layers of a material. This way, 3D printing brings a three-dimensional model into its physical form [14,15,16]. As a result of 3D printing process development, an important number of technologies have appeared. From a pharmaceutical and medical point of view, some 3D printing technologies have relevance. Those whose goals are medical issues (implants, printable organs, etc.) are encompassed as bioprinting [17,18]. Bioprinters are thought with the purpose of being suitable for working with cells and biocompatible materials. These “3D bioprinting technologies” can also be used for pharmaceutical systems [19], in addition to other areas like nourishment [20], which have similar requirements [1].

Natural products and more concretely natural polymers have been extensively employed as pharmaceutical excipients, due to their favorable properties, such as good safety profile, biocompatibility, biodegradability and their origin in renewable sources, in contrast with traditional polymers derived from petroleum, which have an exhaustible nature [21]. Moreover, natural products are also widely employed in tissue engineering, mainly in the form of hydrogels, and are broadly utilized with 3D printing technologies, since they do not need high temperature or organic solvents in their printing process, which makes them suitable for live-cell printing [22]. In general, the requirements that should fulfil natural products for 3D printing applications in tissue engineering and pharmacy include printability, biocompatibility, degradability, tissue biomimicry and appropriate mechanical properties, among others [1].

**Figure 1:**
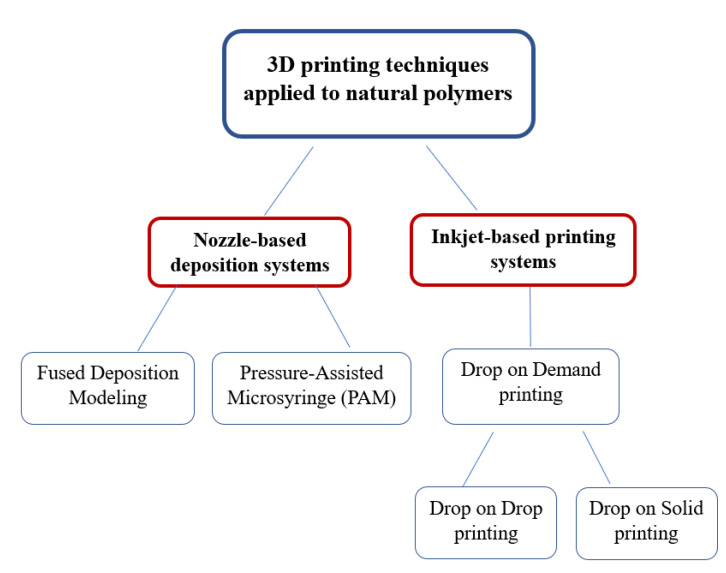
Classification of 3D printing technologies used for natural products.

In this review, the 3D printing technologies employed for the preparation of drug delivery systems based on natural products, which include nozzle-based deposition systems and inkjet-based printing systems, are described. Moreover, a summary of the 3D printed drug delivery systems containing natural products reported in the literature is also included. These drug delivery systems comprise pharmaceutical formulations and drug loaded scaffolds intended for tissue engineering. A discussion is provided about the advantages offered by the natural products to the 3D printing techniques, and the possible reasons why pressure-assisted microsyringe is the most commonly 3D printing technique employed for the obtaining of these drug delivery systems. The 3D printing technologies employed for manufacturing drug delivery systems containing natural products are outlined in Figure 1.

## 2. Nozzle-Based Deposition Systems

These systems are based on the extrusion of material through a nozzle. The material is extruded on the building plate layer by layer in order to build the structure designed with the software. A robotic hand with the nozzle builds in X and Y directions (horizontal plane) and Z direction (vertical plane). Once a layer is finished, the building plate or the printer head move on the vertical axis (Z direction), allowing the extrusion of the next layer with strands of material [23,24,25]. Depending on the 3D printer software, the number of parameters varies. However, there are some parameters which are a basic part of all nozzle-based deposition systems: (i) the nozzle diameter, which affects the diameter of the strand deposited in the building plate; (ii) the feeding rate, the units of which are millimeters (mm) or cubic centimeters per second (cm^3^·s^−1^), and it also defines the number of strands extruded per second. This parameter influences the resolution of the structure (layer thickness) [26]; (iii) the infill percentage, which implies the percentage of extruded material present in each layer, defining the final density. For example, an infill percentage of 100% means that there are no pores at all in this structure that are attributable to the print settings. However, pores may exist, due to the nature of the material used; (iv) the printing speed (mm/s), which refers to the speed of the robotic hand movements during the extrusion [27].

**Figure 2:**
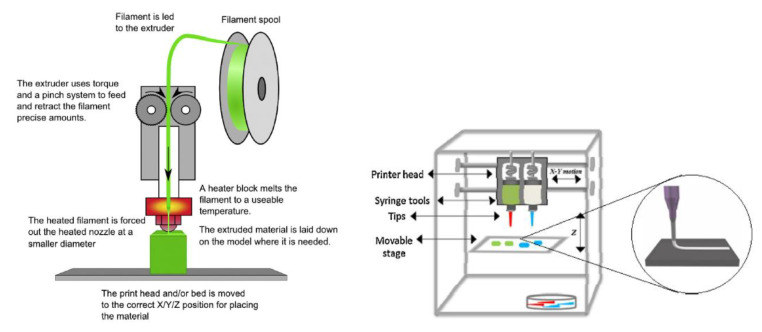
Schematic representation of fused deposition modeling (FDM) (**left**) (from Khatri et al. [2], with permission) and pressure-assisted microsyringe (PAM) (**right**) systems (from Goole and Amighi, [16], with permission)

Nozzle-based deposition systems encompass two main groups: pressure-assisted microsyringe (PAM) and fused deposition modeling (FDM). In the first system, the material is extruded through the nozzle syringe thanks to a pneumatic mechanism, a piston or a screw, which provides the material to build the structure [28] (see Figure 2). In FDM, the extrusion process entails nozzle heating, in order to melt and deposit the material in the building plate [29].

### 2.1. Pressure-Assisted Microsyringe (PAM)

PAM technique is used with pastes, polymer solutions and dispersions. Using this technique, there is no need to increase the temperature for printing the designed structures. As well as in FDM, the drug is incorporated before extrusion [30]. In the last three years, the PAM technique has been employed in 8 studies with natural products to obtain drug delivery systems [31,32,33,34,35,36,37,38]. These studies have been classified according to the nature of the natural product in 3D printed systems employing polysaccharides, proteins and lipids. The particular printing conditions, as well as the dimensions of the scaffolds obtained in the different reviewed works, are shown Table 1.

#### 2.1.1. PAM Technique Employing Polysaccharides

The ability to control the drug release of chitosan was employed by Deng et al. [31] to develop polylactic-coglycolic acid/nano-hydro-xyapatite (PLGA/nHA) scaffolds containing chitosan/recombinant human bone morphogenetic protein 2 (rhBMP-2) nano-sustained release carriers as tissue engineered bone for repairing large jaw defects. Chitosan is a natural product obtained from the shells of crustaceans such as shrimps and crabs. It is a polysaccharide comprising copolymers of glucosamine and *N*-acetylglucosamine obtained by the partial deacetilation of chitin. It is therefore a cationic polyamine that reacts chemically with anionic systems. Chitosan is generally regarded as a nontoxic and nonirritant agent being biocompatible and biodegradable. It is processed in several dosage forms including gels, films, beads, microspheres, tablets and coatings for liposomes, with diverse applications that include immediate drug release, controlled drug delivery systems, such as colonic drug delivery, and mucoadhesive dosage forms [39].

The in vitro sustained release of rhBMP-2 was tested, and its osteogenic effect in rabbit mandibular defects was evaluated. Chitosan nanospheres loaded with rhBMP-2 prepared by the ion-crosslinking method were employed to formulate CS/rhBMP-2 nano-sustained release carriers by adding chitosan acetic acid solution and β-glycerophosphate sodium solution, so the chitosan nanospheres were imbibed in a chitosan hydrogel.

A lyophilized solution of PLGA:nHA in a proportion 4:1 was placed in the A barrel of a 3D bioprinter. The CS/rhBMP-2 nano-sustained release carrier was then loaded into the B barrels. The layers were printed to obtain a PLGA/nHA/CS/rhBMP-2 scaffold complex. A control scaffold without the CS/rhBMP-2 nano-sustained release carrier was also printed, to act as a control material.

It has been observed that the burst effect of the rhBMP-2 at the applied location is responsible for the main side effects of the molecule. Moreover, the timing of rhBMP-2 bioavailability has proved to be important for several days after surgical treatment. The PLGA/nHA/CS/rhBMP-2 scaffold showed good biocompatibility, since no significant inflammatory response was observed in the osteogenic area or surrounding tissues. It also showed biodegradable properties, and was able to control the early burst effect of rhBMP-2. Additionally, the scaffold showed ability to repair experimental bone defects of rabbit mandible, and produced satisfactory osteogenic effects.

The controlled release properties of chitosan were also employed together with alginate to produce a tissue scaffold loaded with drug and cells employing natural polymers for the first time [32]. Alginates are natural polysaccharide polymers isolated from brown seaweed [40]. Sodium alginate is the sodium salt of alginic acid, which is a mixture of polyuronic acids composed of residues of D-mannuronic acid and L-guluronic acid. Due to their recognized lack of toxicity, these polymers have a wide potential to be employed in different types of drug formulations and as food additives. It is used in a wide range of oral dosage forms, such as tablets and capsules. In topical formulations it is used as a thickening and suspending agent in a variety of pastes, creams, gels and as a stabilizing agent for oil-in-water emulsions. Moreover it is used for the aqueous microencapsulation of drugs as well as the formation of nanoparticles [39,40].

This scaffold consists of chitosan coated ionically crosslinked alginate hydrogel fibers loaded with diclofenac and bone cells. These scaffolds were compared with scaffolds without chitosan coating (control sample). The scaffolds were co-cultured with macrophages stimulated to release proinflammatory compounds.

The reported work demonstrated that the chitosan coating increased three times the amount of diclofenac loaded in the scaffold in comparison with the control. Moreover, the coated sample exhibited a slower release pattern, due to the existence of a crosslinked network between the two polymers (the positive group (NH^+^) of the chitosan molecules form ionic crosslinking with the negative group (COO^−^) of the alginate molecules), which acted as a barrier for the drug delivery. In addition, the chitosan coating protected the embedded bone cells from damaging inflammatory compounds produced by stimulated macrophage cells, whose levels were significantly lower. Furthermore, the bone cells in the coated sample showed a higher degree of mineralization and expressed genes that produce proteins for extracellular matrix remodeling in a higher level. It can be concluded that the chitosan coating significantly improved the properties of the studied scaffold, in order to be employed for bone regeneration.

Chitosan was also employed by its gelling capacity in the obtaining of sintering free biphasic calcium phosphate (BCP)/chitosan composite scaffolds containing high solid loadings for bone regeneration and drug delivery [33]. Inks were prepared with 45% *v/v* of BCP powders containing different hydroxyapatite (HA)/β-tricalcium phosphate (β-TCP) ratios, mixed with chitosan solution using a planetary centrifugal mixer. It was intended to maximize the solid loadings while ensuring suitable extrusion behavior. A total of 1% *w/w* of genipin (based on the mass of chitosan) was added to the mixture as a crosslinking agent, followed by homogenization. A total of 2% *w/w* of levofloxacin (based on the mass of inorganic component) was added to the 3% *w/w* chitosan solution, before mixing it with BCP powder in order to obtain levofloxacin-loaded scaffolds, thanks to the non-existence of sintering step.

Scaffolds were 3D printed layer by layer at ambient temperature and humidity. After printing, samples were placed at 37 °C overnight with controlled humidity (80%), to promote chitosan crosslinking by genipin and, subsequently, lyophilized.

In vitro drug release of levofloxacin was carried out in PBS buffer (pH 7.4) at 37 °C and shaken horizontally. At pre-determined time points, samples were collected and analyzed, revealing a high burst release within the first 30 min. Bacteria growth inhibition ability was also shown by levofloxacin loaded scaffolds, demonstrating that the bactericidal effect of the drug was maintained during the fabrication process.

The addition of levofloxacin together with the presence of higher amounts of β-TCP in the BCP composition produced changes in the rheological behavior of the inks and, subsequently, in the morphological and mechanical properties of the scaffolds. Thus, inks with higher amounts of HA were more appropriate to develop calcium phosphate-based scaffolds, with simultaneous activity on infection and bone regeneration.

Chitosan was also employed by Long et al. [34], in this case physically crosslinked with pectin polysaccharides, to obtain hydrogel wound dressings for lidocaine hydrochloride (LDC) delivery. The main property of pectin, which is its ability to form gels based on the formation of hydrogen bonds and hydrophobic interactions between polymer molecules, was taken advantage of. Pectin is a high-molecular-weight, carbohydrate-like plant constituent, obtained mainly from the skin of citrus fruits. It consists of repeating units of D-galacturonic acid linked as 1,4-α-glucosides, with a molecular weight of 30,000–100,000 [39]. It is biocompatible and mucoadhesive. Chitosan and pectin are usually employed crosslinked into polyelectrolyte film or networks to form inserts, microgels, nanocarriers, buccal patches and tablet coatings for colonic drug delivery [41].

The hydrogels were prepared at 0, 2, 5 and 10% *w/w* LDC concentration by mixing pectin and chitosan solutions in HCl at a ratio 1:4. LDC-loaded hydrogel samples were prepared dissolving LDC in pectin solution prior to blending with chitosan solution. The mixture was then transferred to 3D printing syringes and cooled to room temperature to form the gel structure.

The scaffolds consisting of a cubic mesh were fabricated with chitosan-pectin hydrogels with and without LDC using a 3D printer. After printing, the samples were frozen and subsequently lyophilized. The prepared scaffolds showed good printability and good physical integrity, as well as flexibility and self-adhesion to the skin. Swelling and water absorption tests were carried out to evaluate the ability of the lyophilized printed hydrogels to absorb exudates and maintain a moist environment in the wounds. High equilibrium swelling ratios, as well as water absorption values were obtained, demonstrating the good properties of the wound dressings.

In vitro drug release studies showed a burst release of LDC in the first hour, followed by a sustained release of the drug for the following 4 h. The analysis of the release kinetic data demonstrated the fit to the Korsmeyer-Peppas model, with erosion being the mechanism that dominates the drug release. The work reported demonstrates the suitability of the 3D printing technique to prepare customized hydrogels based in natural products for wound dressing.

**Figure 3:**
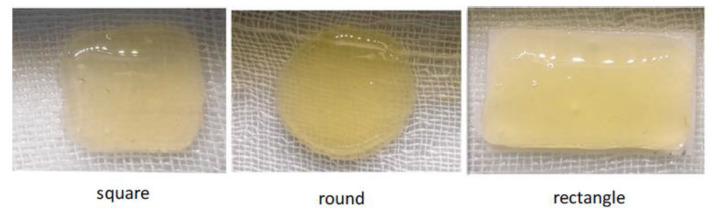
Three different shapes of polysaccharide hydrogel tablets for 3D printing (from Yang et al. [35], with permission).

Other polysaccharides less commonly employed in the manufacture of drug delivery systems, such as *Snakegourd root* and *Astragalus root*, derived from the Chinese traditional medicine, have also been employed by the PAM 3D printing technique. These products show properties such as anti-tumor, immunoregulation and hypoglycemic activity, and were employed by Yan et al. [35], crosslinked with carboxymethyl chitosan (CMC) to prepare hydrogels with different shapes (square, circle rectangle (see Figure 3)) intended to treat diabetic ulcers.

The hydrogels were prepared by mixing a 1:1 volume ratio of 3% *w/w* oxidized polysaccharide solution and different proportions of CMC solutions at room temperature with gentle stirring. The solution was transferred to the 3D printer before the hydrogel gelation. Bovine serum albumin (BSA) was chosen as a model protein to study the release of biomacromolecules from the hydrogels. BSA was dissolved into CMC queous solution, before mixing it with the polysaccharide solution to form the BSA loaded hydrogels in order to study the influence of the hydrogel shape on the drug release rate.

The hydrogels were successfully printed in the three different selected shapes showing appropriate pore structure, swelling behavior and degradation properties. Biocompatibility of the hydrogels was also demonstrated, as well as their suitable rheological properties. Drug release studies were conducted over 168 h for hydrogels placed in Eppendorf centrifuge tubes in a shaker with 5 mL of PBS at 37 °C and pH 1.2 and 7.4. It was observed that the hydrogel with circular shape showed a higher percentage of drug release after 7 days for the two pH values. Additionally, it was demonstrated that hydrogels with higher contents of CMC exhibited a slower drug release, probably due to the higher degree of crosslinking underwent by these samples.

It has been proved that 3D printing technology can be employed to prepare hydrogels with different shapes that can be adapted to the complex and diverse form of the diabetic ulcers of individual patients, demonstrating the great potential that this technology offers in the area of personalized medicine. The employment of natural products enhances the biocompatibility of these preparations, showing great potential for future applications.

#### 2.1.2. PAM Technique Employing Proteins

Gelatin is a widely used excipient, especially known by its utilization in hard or soft capsules. This product consists of a mixture of protein fractions obtained by either partial acid or alkaline hydrolysis of collagen extracted from animal tissues such as skin, sinews and bone. Gelatin has several applications in pharmaceutical technology, like coating, film-forming, gelling or viscosity-increasing agent, among others [39]. This natural polymer may be considered as a nontoxic and nonirritant material, and it is used in oral and parenteral products.

Etxabide et al. [36] have taken advantage of its gelling properties to form a suitable ink to be extruded through a PAM based printer to develop scaffolds loaded with dexamethasone sodium phosphate. A 10% *w/v* gelatin colloidal solution was selected, due to its behavior as a low-viscous liquid during extrusion and as a shape stable gel after ink deposition. Therefore, the viscosity of the gelatin solution was adequate to achieve a continuous filament and control the shape of the printed scaffolds. The solution was prepared with 20% *w/w* lactose as plastizicer and crosslinking agent, and drug (4 mg/mL). In vitro drug release studies of gelatin scaffolds showed the capability of releasing dexamethasone, which is especially important to minimize toxic side-effects caused by the extensive or long-time use of the glucocorticosteroid. Hence, the benefits of gelatin combined with 3D printing technology result in the development of targeted drug delivery biomaterials, reducing the risk of systemic side effects.

#### 2.1.3. PAM Technique Employing Mixture of Polysaccharides and Proteins

Chen et al. [37] have made use of different natural products to prepare PAM 3D printed composite scaffolds coated by layer-by-layer (LBL) deposition intended for bone tissue engineering. Concretely, sodium hyaluronate and chitosan were employed to coat by LBL porous hydroxyapatite (HAP) scaffolds manufactured by PAM bio-printing. These scaffolds were prepared from HAP nanoparticles, gelatin and hyaluronate solution.

Sodium hyaluronate is the sodium salt of hyaluronic acid, a glycosaminoglycan constituted by D-glucuronic acid and *N*-acetyl-D-glucosamine disaccharide units. It is present in vitreous humor, serum, chicken combs, shark skin and whale cartilage. It can be extracted from its natural source, but it may also be manufactured by fermentation of selected *Streptococcus zooepidemicus* bacterial strains. It is used in the treatment of the knee osteoarthritis and it is effective in the relief of arthritic pain. It also enhances the availability and retention time of drugs administered to the eye [39]. Its immunoneutral properties make possible its use for the attachment of biomaterials in tissue engineering.

These scaffolds were prepared by mixing HAP nanoparticles with gelatin and hyaluronate solution using a planetary mixer. A slurry with an adequate viscosity was obtained, thanks to the viscosity change that gelatin experiences at 37 °C, which increases its adhesiveness to HAP. Moreover, sodium hyaluronate was employed to adjust the viscosity of the slurry for 3D printing. The slurry obtained this way was transferred to the 3D bio-printing system. The strands were extruded by air-pressure. The composites solidified after extrusion because of the drop of temperature. After that, the 3D printed scaffolds were cross-linked in glutaraldehyde solution, washed with de-ionized water and freeze-dried in a lyophilizer.

**Figure 4:**
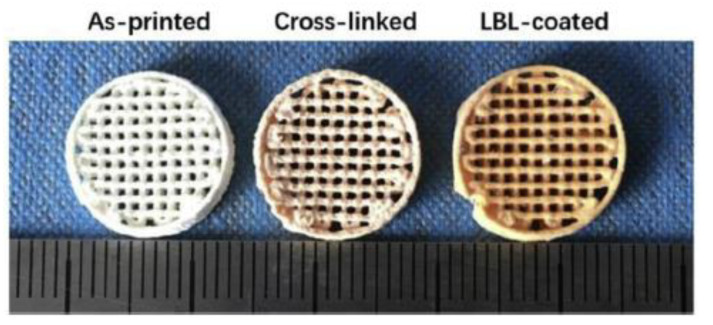
Photographs of an as-printed, cross-linked and coated scaffold (from Chen et al. [37], with permission).

Sodium hyaluronate and Chitosan multi-layers were deposited onto the scaffolds, by immersing them in Sodium hyaluronate and Chitosan solutions repeatedly, obtaining scaffolds with 10 and 20 bilayers. The coated scaffolds were freeze-dried, and were stored in a desiccator for further studies. Figure 4 shows the aspect of the as-printed, cross-linked and coated scaffolds.

After that, the scaffolds were loaded with Rhodamine (RHB) and Bovin Serum albumin (BSA) by immersing them in a solution of each component. These molecules were selected as model drugs to simulate antibiotics and growth factors, respectively. The loading efficiency of BSA decreased after coating, probably because of its high molecular weight, indicating that if it is needed to load high molecular weight growth factors or drugs they must be loaded before LBL coating.

RHB released a little slower in the LBL coated scaffolds in comparison with the non-coated scaffold. On the contrary, BSA showed a faster release rate for the LBL coated scaffolds. The LBL coating is supposed to reduce the swelling ratio of scaffolds, which is beneficial since swelling could result in damaging of the surrounding tissues. Moreover, the LBL coating increased the compressive strength of the scaffold around 70% and decreased its degradation rate.

Scaffolds with LBL coating showed good biocompatibility with MC-3T3E1 cells, and provided proper conditions for cell adhesion and proliferation, indicating that the printed hydroxyapatite composite scaffolds exhibit a great expectative as candidates for bone repair and as carriers for drug and growth factors.

#### 2.1.4. PAM Technique Employing Lipids

Until now, no chocolate-based oral formulations are marketed, although there have been studies that demonstrate its efficacy, safety and tolerability in children [42]. Traditionally, *Theobroma oil*, the major ingredient of chocolate, has been used as a suppository base taking advantage of its melting point close to physiological temperature (31–34 °C). However, due to problems associated to the formation of metastable forms during the preparation, this substance was displaced by other hard fat suppository bases [39]. Regarding oral formulations, chocolate has been previously evaluated to mask the bitter taste of drugs, improving the treatment acceptability by the pediatric population [43,44,45].

As has been mentioned, 3D printing technology offers the opportunity to make customizable design of dosage forms. In the case of pediatric population, a more attractive drug product can result in higher patient compliance and treatment adherence. Pediatric-appropriate formulations must fulfill more requirements than those for adults, such as: high variability of dosage strength according to age/weight, ease of administration or taste masking. Hence, the liquid dosage forms are very frequently prescribed for this therapeutic group. However, it is well known that these formulations are related to problems of dosing inaccuracy or instability, among others.

**Figure 5:**
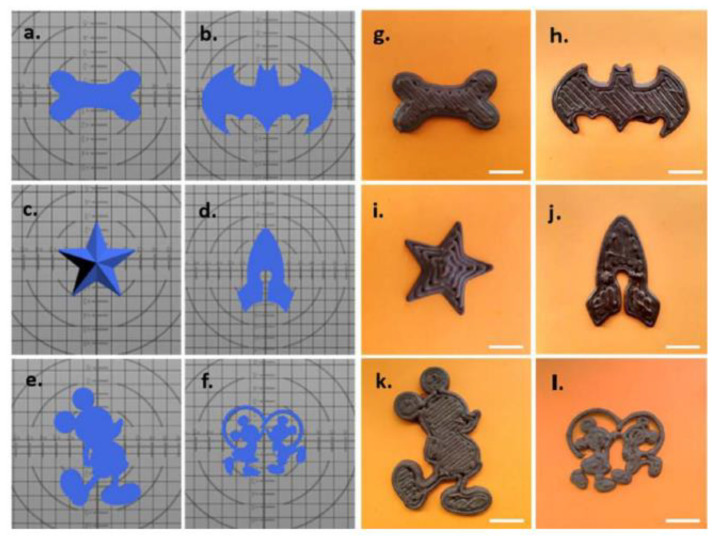
Schematic representation of the (**a**–**f**). Stl files and the (**g**–**l**). 3D printed chocolate-based dosage forms. Scale bar: 20 mm (from Karavasilli et al. [38], with permission).

Recently, a pediatric-friendly chocolate-based formulation has been developed by 3D printing technology [38]. In that work, varied chewable dosage forms with both hydrophobic and hydrophilic drugs were obtained by PAM 3D printing. Drug loaded chocolate inks were easily prepared by melting bitter chocolate and adding corn syrup containing dissolved paracetamol or melted ibuprofen, at a final concentration of 22.9 mg/g and 19.6 mg/g, respectively. These inks were immediately loaded in the cartridges of the 3D printer and heated up to 45 °C. Then, the inks were printed according to different designs as a star or cartoon characters (see Figure 5).

Physicochemical and rheological studies were performed to evaluate the main properties of the chocolate-based inks and the drug-loaded formulations. The chewable chocolate-based dosage forms showed a fast and high drug dissolution in simulated saliva fluid. Hence, the combination of the customization provided by 3D printing technology and the palatability of the natural product chocolate results in an attractive dosage form for the pediatric population.

### 2.2. Fused Deposition Modeling (FDM)

The main difference between FDM and PAM are the physical properties of the material employed: for the FDM technique, plastic filaments are needed because of their capability to melt at certain temperature. Generally, hot melt extrusion (HME) is the previous step before FDM, and it is utilized to obtain the strands, thanks to a hot melt extruder that mix thermoplastic polymers in form of powder or pellets. In pharmaceutical field, the drug is added together with plasticizers and a suitable polymer to obtain filaments charged with the active compound [46]. The particular printing conditions, as well as the dimensions of the scaffolds obtained in the different reviewed works are shown in Table 2.

This technique has been employed with two biopolymers: chitosan and alginate. With respect to chitosan, Eleftheriadis et al. [47] took advantage of its properties as penetration and mucoadhesion enhancers in the preparation of poly(vinyl alcohol)-based mucoadhesive films intended for unidirectional release of the model hydrophilic drug diclofenac sodium (DNa). The polymeric filaments were prepared employing poly(vinyl alcohol), due to its suitable properties as a film forming agent, its high thermo plasticity and its excellent printability, xilytol as plasticizer and DNa as model drug. The effect of chitosan on mucoadhesion and drug permeation was investigated preparing the filaments with and without 1% *w/w* of the natural polymer.

In order to investigate the effect of a backing layer to obtain unidirectional release profile, ethyl cellulose (EC) with triethyl citrate as plasticizer in a 90:10% *w/w* proportion and commercial wafer edible sheet (WAF) were evaluated. The PVA and EC filaments were produced by a single screw extruder. Films without a backing layer were 3D printed. For the EC backing layer, a square item was printed on the top surface of the drug loaded films. When WAF was employed as backing layer it was applied on the building platform. Thus, the addition of chitosan to the formulation demonstrated enhanced mucoadhesion and permeation properties. Moreover, the presence of a baking layer resulted in modified release profiles with unidirectional release of DNa.

Gioumouxouzis et al. [48] also employed chitosan to prepare drug delivery systems by FDM 3D printing, taking advantage of its ability to provide a sustained release. The authors also utilized sodium alginate as biopolymer for the elaboration of a hollow pH responsive FDM 3D printed tablet for targeted colonic delivery. The capacity of the sodium alginate of microencapsulating drugs is employed in this work, to prepare non-coated and chitosan-coated alginate beads containing 5- fluorouracil (5-FU), which were encapsulated in the printed tablet.

The formulation prepared is constituted by an insoluble PLA upper compartment and a bottom layer consisting of a mixture of polymethacrylates soluble in the colonic pH. The pH responsive layer of the tablet was manufactured from filaments containing different combinations of Eudragit^®^ L100-55/S100 and triethyl citrate (TEC) as plasticizer, which were prepared employing a single-screw extruder. Printing was carried out in a 3D printer with two nozzles using the first nozzle for printing the Eudragit^®^-based lower layer and the second nozzle for printing the PLA upper compartment.

The 5-FU alginate beads were loaded into the formulation, pausing the printing before completion, and distributing them into the hollow part of the PLA compartment (30% infill). The release of 5-FU alginate at pH values corresponding to the colonic segment of the gastrointestinal tract was shown by the in vitro release studies. Non-coated alginate beads showed a faster 5-FU release from the 3D printed dosage forms, although differences were not statistically significant. This innovative dosage form combines the advantages of the multiparticulate dosage forms with the manufacturing versatility of 3D printing technology for creating personalized medicines, which deserves further investigation for the treatment of colorectal cancer, as well as other pathologies.

#### HME Filaments Containing Natural Products as APIs

The 3D printed medicines containing natural products as API have not been developed yet. Nevertheless, there are some studies that employ plant extracts to manufacture filaments by hot-melt extrusion [49,50,51,52]. The possibility of obtaining filaments loaded with plant extracts offers a valuable potential for the preparation of 3D printed drug delivery systems containing natural APIs by FDM.

Pinho et al. [49] produced filaments containing cocoa extracts (CE) obtained from *Theobroma cacao*. This natural product, commonly employed in the food industry, also shows therapeutic potential, because of its cardioprotective and anti-inflammatory actions [53]. Different combinations of three hydrophilic polymeric matrices (Soluplus, Plasdone S630, and Eudragit E) were employed to obtain the filaments with a proportion CE-polymer 3:7 (*w/w*). A single step hot-melt extrusion (HME) process was employed. The reported study concluded that the natural product stability was preserved, as well as the presence of drug-polymer interactions.

On the other hand, filaments containing *Ginkgo biloba* extracts (GBE), which are widely employed to treat cardiovascular and neurodegenerative diseases, were prepared by Wang et al. [50] employing also a HME process. In this case, a solid dispersion of GBE was firstly prepared employing as matrix carriers a Kollidon^®^ VA64/Kolliphor^®^ RH40 (85:15) spray dried powder. GBE and the matrix carriers were premixed obtaining physical mixtures that were processed via HME, obtaining extrudates containing 25% (*w/w*) GBE.

*Angelica gigas Nakai* (AGN), a popular herbal medicine used in Asian countries for its anticancer, anti-amnestic and anti-allergic effect, has also been employed to obtain filaments by HME. In the work reported by Jiang et al. [51], the fresh root of AGN was dried in an oven. The dried sample was powdered by a pin crusher obtaining a coarse powder that was pulverized. The ultrafine powders alone, as well as the ultrafine powders mixed with Soluplus were extruded, employing a hot melting extruder.

Curcumin, a coloring and biologically active constituent of *Curcuma long* L., was employed by Chuah et al. [52] in the preparation of filaments by HME. The aim of this study was to obtain a functional food ingredient, due to the wide therapeutic properties, such as anticancer, antiviral or anti-inflammatory effects that this component shows. Concretely, 10% (*w/w*) curcumin powder, 75% (*w/w*) HPMC, 10% (*w/w*) lecithin and 5% (*w/w*) isomalt were blended and subjected to HME, using a co-rotating twin screw extruder to obtain filaments of an amorphous solid dispersion.

## 3. Inkjet-Based Printing Systems

This term encompasses those systems that work with digitally controlled formation and placement of small liquid droplets. They can be divided into continuous inkjet (CIJ) printing and drop on demand (DoD) printing. This review will focus on DoD printing, because it is widely used for research purposes. CIJ employs much more ink than DoD; in addition, the ink can be degraded by the environmental exposure that takes place during the recycling ink process, thus, it is not a suitable technique for the development of 3D drug delivery systems [54,55].

DoD printing generates small droplets (1–70 picolitre) using two different techniques: piezoelectric ceramic pieces and thermal inkjet heads. Both are located close to the printhead, expelling the drop only when necessary. This is another difference with respect to CIJ. Regardless of the inkjet drop generator used: inertia, surface tension and drop viscosity play key roles during the printing process [56].

Piezoelectric ceramic pieces generate drops thanks to the deformation suffered as a result of an electric signal which pass through them. The deformation pushes a volume of ink to the printhead, resulting in drops generation. On the other hand, thermal inkjet heads are restricted to inks that can vaporize in specific conditions. As a result of the vaporization process, the bubble expansion pushes the ink through the printhead [55].

**Figure 6:**
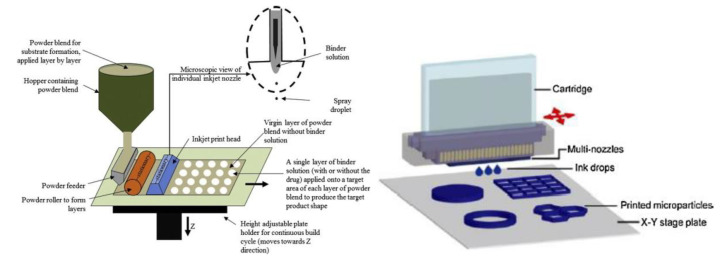
Illustration of drop on solid (**Left**) and drop on drop (**right**) printing process (from Norman et al., [4] and Goole and Amighi, [16], with permission).

There are different techniques below the umbrella term of DoD printing (Figure 6): drop on solid printing and drop on drop printing. Drop on solid printing deposits a liquid drop in a powder bed according to a pattern. Once a layer is finished, a roller places a new powder bed ready to be linked. This is a very common technique in the pharmaceutical area [57,58]. As an example, Aprecia Pharmaceuticals employs it for its formulation Spritam^®^ (levetiracetam), becoming the first 3D printing technology medicine approved by the Food and Drug Administration (FDA) [59].

As stated before, another type of technique included in DoD printing is drop on drop printing. In this technique, once a drop is dispensed, a thermal stimulus from the building plate causes solvent evaporation and polymer solidification, allowing the deposition of a second drop. Droplets overlapping makes possible to build 3D structures with high resolution [16,60]. The particular printing conditions, as well as the dimensions of the scaffolds obtained in the different reviewed works, are show in Table 3.

The hydrophobic character of beeswax has been employed by Kyobula et al. [60] to be combined with insoluble drugs, such as fenofibrate, to control its release profile, making use of an inkjet-based 3D printing approach, concretely drop on drop printing. Beeswax is a natural wax obtained from honeycombs made by the honeybee, *Apis mellifera*. Chemically, this wax consists of 70–75% of a mixture of various esters of straight-chain, monohydric alcohols with even-numbered carbon chains from C24 to C36, esterified with straight-chain acids [39]. Beeswax is regarded as nontoxic and nonirritant material, recognized as GRAS (Generally Recognized as Safe) by FDA. Its use is accepted in both topical (especially in ointments, creams and water-in-oil emulsions) and oral formulations. Regarding oral formulations, this ingredient can be used in tablets or nanoparticles as polishing or sustained-release agent. In a study by Kyobula et al. [60], beeswax was employed for the first time to manufacture a customizable pharmaceutical dosage form by 3D inkjet drop on drop printing. The use of an inkjet approach allows for the manufacture of complex formulations with high resolution geometries, evaluating the effect of architecture on drug release profiles. In this work, a solvent free ink was investigated using mixtures of 5% *w/w* fenofibrate, and melted beeswax as drug carrier. This ink was immediately placed in the piezoelectric inkjet printer with hot melt chamber and printed at 90 °C. Therefore, all the process was carried out without the need of solvent, which eliminates the toxicological risks associated to them.

The hydrophobic character of beeswax has been employed by Kyobula et al. [60] to be combined with insoluble drugs, such as fenofibrate, to control its release profile, making use of an inkjet-based 3D printing approach, concretely drop on drop printing. Beeswax is a natural wax obtained from honeycombs made by the honeybee, *Apis mellifera*. Chemically, this wax consists of 70–75% of a mixture of various esters of straight-chain, monohydric alcohols with even-numbered carbon chains from C24 to C36, esterified with straight-chain acids [39]. Beeswax is regarded as nontoxic and nonirritant material, recognized as GRAS (Generally Recognized as Safe) by FDA. Its use is accepted in both topical (especially in ointments, creams and water-in-oil emulsions) and oral formulations. Regarding oral formulations, this ingredient can be used in tablets or nanoparticles as polishing or sustained-release agent. In a study by Kyobula et al. [60], beeswax was employed for the first time to manufacture a customizable pharmaceutical dosage form by 3D inkjet drop on drop printing. The use of an inkjet approach allows for the manufacture of complex formulations with high resolution geometries, evaluating the effect of architecture on drug release profiles. In this work, a solvent free ink was investigated using mixtures of 5% *w/w* fenofibrate, and melted beeswax as drug carrier. This ink was immediately placed in the piezoelectric inkjet printer with hot melt chamber and printed at 90 °C. Therefore, all the process was carried out without the need of solvent, which eliminates the toxicological risks associated to them.

**Figure 7:**
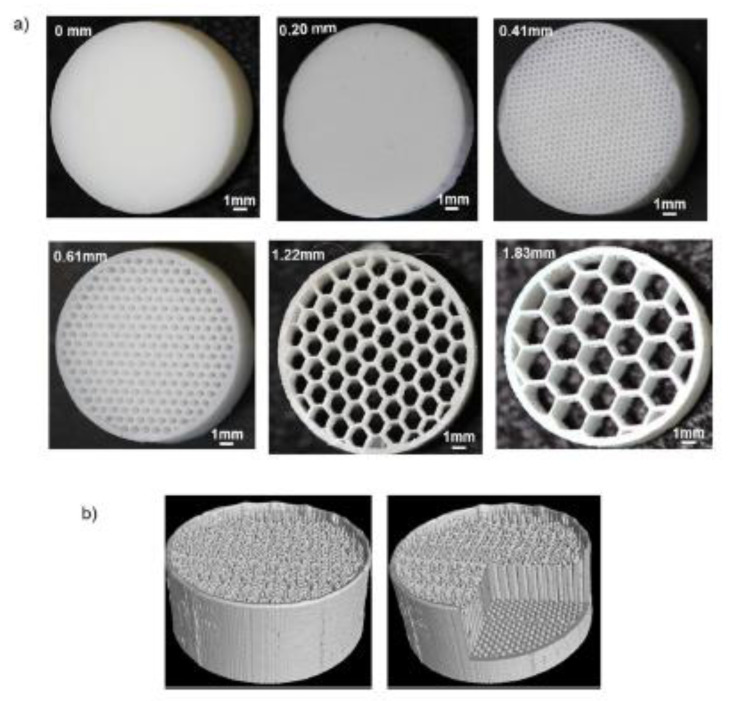
(**a**) Photographs of the printed solid tablet and honeycomb-like tablets with varying cell sizes between 0.20 mm and 1.83 mm; (**b**) 3D micro X-ray computed tomography (μCT) scan images obtained for the honeycomb architecture tablets, the right hand image has a digital cut-away (from Kyobula et al. [60], with permission).

The use of beeswax is not the only element taken from the nature, but also the design of the 3D systems, inspired in the honeycomb architecture. The high resolution of inkjet printing offers the advantage to place accurate volumes of ink and a very precise spatial localization of materials. So, honeycomb systems were printed with different cell diameters (see Figure 7).

According to the surface area exposed to the medium, a higher drug release was expected as the cell diameter decreases. However, researchers found the slowest drug release profile for the smallest cell diameter. This fact was attributed to the poor wetting effect of the hydrophobic beeswax. Biomimetics used in this work makes it possible to modify the drug release profiles without the need to change the formulation.

The fast gelling reaction of chitosan, and its ability to retain drug in microporous implants, together with the inkjet technology, have been exploited by Vorndran et al. [57] to develop microporous bioceramics implants for repairing bone defects, achieving accurate drug deposition.

Localized drug release provides great advantages compared to systemic delivery, decreasing the dose required, the risk of side effects and the cost of treatment. In the particular case of the reconstruction of bones, 3D printing is especially indicated, as the scaffold can be designed to perfectly fit on the anatomy of the patient. Chitosan plays a crucial role on the microporous scaffold as polymer inhibits the drug diffusion in the structure by producing a fast gelling reaction thanks to the reaction of negatively charged polymers and polyanions in contact with the aqueous environment. Consequently, the drug remains in the scaffold, instead of suffering a fast release. The multijet drop on solid 3D printer allows to elaborate calcium phosphate scaffolds at low temperature with an accurate localization of drugs (recombinant bone morphogenic protein 2 (rhBMP-2), heparin and vancomycin) [57]. With the additive manufacturing the drug distribution within the implant structure can also be controlled, loading API in concrete positions: homogeneously dispersed, concentrated in the center or dispersed along a concentration gradient. Hence, researchers could also evaluate the effect of API localization on drug release kinetics [57]. Therefore, the use of biomaterials as the main ink component for 3D printing makes it possible to achieve highly customized degradable bone implants.

## 4. Discussion

Until now, the use of natural substances in the production of pharmaceutical dosage forms by 3D printing technology is still limited. Despite the unquestioned advantages that they offer with respect to synthetic materials, some limitations in the terms of printability make its use difficult. Some of the advantages of the employment of natural products in pharmaceutical formulations obtained by 3D printing, as well as their printability characteristics, are summarized in Table 4.

Obviously, the biocompatible and biodegradable properties of these substances offer a great advantage, especially when they are used in implants. Among natural products, biopolymers stand out for their versatility and photo-curable or thermoplastic properties, which make them suitable for the different additive manufacturing. Moreover, taking the advantage of most of the biopolymers to form matrix systems, they are used to obtain a controlled drug release, particularly in the case of chitosan. In addition, the ability of alginate and chitosan to form hydrogels is exploited in PAM and inkjet techniques, distinguishing chitosan by the fast gelling reaction. Chitosan is also employed physically crosslinked with pectin, to form a hydrogel matrix able to entrap the API by PAM. In this sense, hydrogels are postulated as one of the most suitable classes of ink materials for 3D printing, acquiring an important role in the development of biomedical devices [61]. They have the ability to be fabricated in customized shapes and offer many advantages for cell culture [62] and pharmaceutical formulations [34,35]. Other natural products such as gelatin or sodium hyaluronate are employed, due to their ability to adjust the viscosity of the slurry at body temperature by PAM 3D printing, which is very important in the case of the use of cells in tissue engineering. In addition to the products for the preparation of pharmaceutical formulations and drug loaded scaffolds reviewed here, a wider variety of natural products are being processed, using 3D printing technologies to manufacture medical devices [63,64,65,66].

With regard to the printability, the role played by chitosan should be highlighted. This versatile excipient can not only be found as a component of the 3D printed systems, but also as gelling agent coating the surface of the 3D printed systems. Chitosan is able to gel with negatively charged polymers and polyanions. The proportion of chitosan in the mixture should be low, to avoid the clogging of the nozzle in the inkjet and FDM techniques. However, the gel extruded by PAM requires higher viscosity. Extrudable crosslinked gel with pectin and chitosan was obtained heating and mixing both components at 65 °C. Before the 3D printing process, this mixture had to be cooled up to ambient temperature to obtain an extrudable hydrogel matrix.

In the case of the beeswax, it was necessary to increase the temperature above its melting point, in order to obtain a suitable drop without blocking the nozzle during the printing process. Regarding chocolate, it was melted and mixed with corn syrup to achieve a suitable paste to be extruded, forming an appropriate strand. Regarding gelatin, it is necessary to adapt its viscosity, regulating the temperature and its concentration in the mixture, to achieve a gel capable of controlling the release of the drug and an adequate printing capacity. Alginate is another natural product able to produce a gel with right properties to be printed by PAM, due to the reasons detailed below. *Snakegourd* root, *Astragalus* root and sodium hyaluronate do not have the capability to be printed on their own, but they help to obtain a printable gel. In addition, sodium hyaluronate has been applied as an adjuvant with chitosan to coat the final system.

With respect to the most used 3D printing technique with natural products, it is possible to discern a trend towards the PAM technology. This can be attributed to different reasons. In the field of biomedicine, porous materials, which facilitate new tissue formation, are often sought. These materials should induce matrix formation, cell migration and adhesion, being biocompatible and biodegradable. Natural polymers are postulated as good candidates to satisfy these requirements, since most of them can form hydrogels at different proportions, resulting in porous matrices. However, natural hydrogels need mild conditions to be processed so that not all 3D printing techniques are suitable [61]. PAM is compatible with hydrogels of a wide range of viscosities and can form bioresorbable, bioactive and mechanically robust structures, with precision and flexibility. Nevertheless, FDM possesses the potential to become a suitable candidate to manufacture drug delivery systems containing natural products. Although no work has been found employing FDM for natural products, as mentioned in Section 2.2, some researchers have clearly demonstrated the feasibility of HME to develop filaments made of natural products [49,50,51,52]. Seeing HME as a previous step of FDM, it is reasonable to consider FDM as alternative to PAM.

The main reason why FDM has not yet been widely employed with natural products is probably the processing temperature. During the last few years, FDM has been applied to excipients and APIs that support high temperatures. However, a current trend is growing towards a more versatile application of FDM, decreasing the temperatures and using excipients with low melting point. These milder conditions make the use of FDM possible for thermolabile drugs and natural products [10,67]. Solid dispersion as a method to improve poorly water-soluble drugs, high resolution objects and free-dissolvent methodology are hallmarks of FDM, compared with PAM, thus, it is expectable new researches employing FDM in the near future. In addition, the increasing use of FDM and PAM, named as extrusion-based methods, can be explained by a clear economic motivation, as they are the less expensive technologies and with compact size equipment [68].

Despite the significant increase of the use of biomaterials in 3D printing technology in the last few years, there are important challenges to overcome. One of them is related to the inherent variability of natural products of different sources, which can result in a risk for its utilization. Additionally, there is a need for a complete characterization in terms of the printability of these products like mechanical integrity, visco-elastic properties, in situ gelation, high resolution during printing, etc. The understanding of these aspects will allow a rational selection of the bioink materials for the 3D printing of dosage forms.

## 5. Conclusions

The utilization of natural substances in 3D printed pharmaceutical formulations provides clear advantages related to biocompatibility and biodegradability of the manufactured systems. In the case of application to implants and scaffolds for tissue engineering, these benefits become crucial. Natural products have a wide variety of properties that makes it possible to use them in a large number of 3D printing technologies, including FDM, PAM or inkjet-based printing techniques. Nevertheless, this field is still in its early stages. There are very few research studies reported in the current literature. Furthermore, the use of natural products is restricted to the role of excipient. The incorporation of natural products as drugs, the harnessing of its high affinity to targets with whom they naturally bind, the improvement in the characterization techniques and the utilization of metabolites produced by microbes and plants predict a very promising future for these products in 3D printed systems.

## Figures and Tables

**Table 1:** Printing conditions and scaffolds dimensions of PAM 3D printed systems containing natural products.

Natural Product	Type of Printer Used	Scaffolds Dimensions	Printing Temperature	Printing Speed	Reference
Chitosan	3D bioprinter (Youni Technology Co., Ltd., organization 2500 X, Shenzhen, China	13 × 6 × 4 mm	4 °C	4 mm/min	[31]
Chitosan and alginate	3D plotting system (SR2000D, Ganbow Technology, Banqiao, Taiwan)	10 × 10 × 2 mm	Room temperature	35 mm/s	[32]
Chitosan	Robotic deposition device (3-D Inks, Stillwater, OK, USA)	3 × 3 × 3 mm	Room temperature	10 mm/s	[33]
Chitosan and pectin	BioBot1 3D printer (Allevi, Philadelphia, PA, USA)	23.50 × 23.50 × 1.00 mm	Room temperature	6 mm/s	[34]
*Snakegourd root* and *Astragalus root*	Melt-extrusion 3D printer	Square, circle and rectangle shapes	Room temperature	50 mm/s	[35]
Gelatin	BioBots 3D printer (Allevi, Philadelphia, PA, USA)	22.20 × 11.20 × 0.80	27 °C	4 mm/s	[36]
Sodium hyaluronate and chitosan	3D bio-printing (Regenovo Bio-printer system, Hangzhou Regenovo biotechnology Co., Ltd., Hangzhou, China)	Cylindrical shape (Diameter = 10 mm, Height = 5 mm)	37 °C	8 mm/s	[37]
Chocolate	3D Food Printer (Model 3C10A)	Different shapes ranging from 59.1 × 33.1 × 3.0 mm to 61.8 × 84.1 × 6.0 mm	45 °C	5 mm/s	[38]

**Table 2:** Printing conditions and scaffolds dimensions of FDM 3D printed systems containing natural products.

Natural Product	Type of Printer Used	Scaffolds Dimensions	Printing Temperature	Printing Speed	Reference
Chitosan and alginate	Makerbot Replicator^®^ 2X FDM printer (Makerbot Inc., New York, NY, USA)	20 × 20 × 0.2 mm EC backing layer: 20 × 20 × 0.1 mm	200 °C (PVA) 190 °C (EC)	10 mm/s	[47]
Chitosan	MakerBot Replicator^®^ 2X 3D printer (MakerBot Inc., New York, NY, USA)	Cylindrical shape (Diameter = 15 mm, Height = 4.2 mm)	182 °C (Eudragit^®^) 215 °C (PLA)	20 mm/s	[48]

**Table 3:** Printing conditions and scaffolds dimensions of inkjet-based printed systems containing natural products.

Natural Product	Type of Printer Used	Dimensions of the Scaffolds	Printing Temperature	Reference
Beeswax	Inkjet printer (PiXDRO LP50, Meyer Burger Technology Ltd., Gwatt (Thun), Switzerland )	Cylindrical shape: 0.20, 0.41, 0.61, 1.22 and 1.83 mm diameter and 3.22 mm height	90 °C	[60]
Chitosan	Multicolor 3D powder printing system (spectrum Z510, Z Corporation, Burlington, MA, USA)	----	----	[57]

**Table 4:** Advantages of the employment and printability of natural products in 3D printed pharmaceutical formulations.

Natural Product	3D Printing Technique	Advantages	Printability	References
Beeswax	inkjet	Solvent free ink/Controlled release for hydrophobic drugs	Suitable surface tension and droplet formation at 90 °C	[60]
Chitosan	PAM	Ability to control the drug release Protection of the bone cells imbibed in the scaffold/Increase of the amount of drug loaded in the scaffold	Adequate rheological properties of hydrogels with low viscosity and fast gelling reaction with negatively charged polymers and polyanions	[31,32,35,37]
	FDM	Mucoadhesive properties		[47]
	Inkjet	Fast gelling reaction/Retention of drug in microporous implants		[57]
Chocolate	PAM	Taste masking	No nozzle clogging and correct strand extruded at 45 °C	[38]
Gelatin	PAM	Shape control of structure/Controlled drug release	Extrudable hydrogels with variable viscosity depending on the printing temperature	[36]
		Viscosity change at 37 °C which increase cohesiveness with HAP		[37]
Sodium hyaluronate	PAM	Adjustment of the viscosity of the slurry for 3D printing/Extrusion at body temperature	----	[37]
		Coadyuvant of the controlled release		
*Snakegourd root* and *Astragalus root*	PAM	Ability to form hydrogels/hypoglycemic activity	----	[35]
Alginate	PAM	Very mild gelling condition biologically safe for cells	Ease gelation with low percentage and mild conditions	[32]
		Control of the drug release		
Physically crosslinked Chitosan/Pectin	PAM	Avoidance of chemical crosslinkers typically toxic/entrapment of the drug in the hydrogel matrix	Extrudable gel structure after cooling up at ambient temperature	[34]
